# Land Use Dynamic Evolution and Driving Factors of Typical Open-Pit Coal Mines in Inner Mongolia

**DOI:** 10.3390/ijerph19159723

**Published:** 2022-08-07

**Authors:** Lijia Zhang, Zhenqi Hu, Dazhi Yang, Huanhuan Li, Bo Liu, He Gao, Congjie Cao, Yan Zhou, Junfang Li, Shuchang Li

**Affiliations:** 1 School of Geosciences & Surveying Engineering, China University of Mining and Technology, Beijing 100083, China; 2 Land Consolidation and Rehabilitation Center, Ministry of Natural Resources, Beijing 100035, China; 3 Key Laboratory of Land Surface Pattern and Simulation, Institute of Geographic Sciences and Natural Resources Research, Chinese Academy of Sciences, Beijing 100101, China; 4 College of Resources and Environment, University of Chinese Academy of Sciences, Beijing 100049, China; 5 School of Land and Tourism, Luoyang Normal University, Luoyang 471000, China; 6 School of Geomatics, Liaoning Technical University, Fuxin 123000, China; 7 School of Earth Sciences, Yangtze University, Wuhan 430100, China; 8 School of Earth Sciences and Resources, Chang’an University, Xi’an 710064, China

**Keywords:** open-pit coal mines, GEE, land use, dynamic degree, driving factors, Inner Mongolia

## Abstract

Although coal is difficult to replace in the short term, the large-scale production and consumption of coal have significant impacts on the ecological environment. The severe disturbances, such as land excavation and occupation, that accompany the mining of mineral resources have caused dramatic changes in land cover and a significant pressure on the sensitive and fragile ecological environment. To analyze the temporal and spatial evolution trends and the differences in land use in different typical mining areas in Inner Mongolia, as well as the evaluation system and driving mechanisms of land use evolution, this study takes the typical open-pit coal mines in Inner Mongolia as the research objects and, based on the Google Earth Engine (GEE) platform, analyzes the dynamic evolution characteristics and driving factors of land use in typical open-pit coal mines in Inner Mongolia from 2001 to 2020. The change trend of land use in typical open-pit mining areas in Inner Mongolia for the past 20 years is obvious, with the highest fluctuations for grassland, mining land, cropland, and residential/industrial land. Land use in the open-pit coal mining area is greatly affected by mining factors. From the perspective of spatial variation, the most important driving factor is the distance from national roads and railways, followed by the annual average temperature and annual average precipitation and topographical conditions, such as elevation. In terms of policy, land reclamation and ecological restoration in mining areas have a positive impact on land use change. Improving the mechanism for environmental compensation in mining areas can promote the efficient and rational use of mining areas and the protection of ecosystems.

## 1. Introduction

Coal is the world’s largest and most widely distributed non-renewable energy, and plays an irreplaceable role in the development of the national economy [1]. Against the background of climate change, since the 21st century, greenhouse gas emission reduction, carbon neutrality, and the adjustment of energy structure have received significant attention, and numerous developed countries have progressively adopted clean energy to replace coal energy consumption [2,3]. However, in developing countries, especially China, India, South Africa, and Indonesia, among others, coal resources are still the most important energy sources [4,5,6]. China’s coal production far exceeds that of other countries, accounting for about 51% of the global coal production [7], and the mining industry has brought improved infrastructure, economic development, and elevated living standards for locals [8,9]. In most countries, coal resources are still the most important energy source for power and heat generation [1], and the principal raw material for various daily necessities, such as dyes, fertilizers, and pesticides [10].

However, the large-scale production and consumption of coal have seriously negatively impacted the ecological environment, with an emphasis on land resources, by directly destroying the surface soil layer and original vegetation [11,12,13]. Wastewater from mining is generally discharged into rivers, resulting in the death of aquatic animals and plants and the destruction of river ecosystems [14]. The infiltration of acidic wastewater into the ground also leads to the deterioration of groundwater quality and the destruction of the vegetation. In addition, excessive mining activities produce large amounts of carcinogenic heavy metals that are difficult to degrade in the natural state, such as chromium, nickel, cadmium, with negative impacts on human and animal health [15,16].

Open-pit mining is the most commonly used method of coal mining because of its low cost and convenience; two thirds of the world’s mineral resources are extracted via open-pit mining [17,18]. More than 50% of coal resources in the United States, Australia, Spain and other countries are mined in open-pit mines [19,20]. However, because of the “stripping-mining-transportation-disposal-land making” in the mining area, the original ecosystem is predominantly degraded, with deteriorated surface water and groundwater and a diminished carbon storage capacity [21,22,23]. According to the data released by the National Research Council (NRC) of the United States, open-pit mining of 124 billion tons of coal in the United States will destroy about 4 million hm^2^ of land. Under the same mining volume, China will destroy 2.728 million hm^2^ of land [24,25,26]. The severe disturbances, such as land excavation, compaction and occupation during the mining of mineral resources, have caused dramatic changes in the land cover in this area [27], resulting in altered ecosystem types, patterns and processes and, ultimately, in changes in ecosystem services [28]. In this sense, studies on land use change in open-pit coal mining areas are valuable to assess the evolution of the ecosystems in such areas [29].

In China, more than 90% of large open-pit coal mines are located in arid and semi-arid areas with a fragile ecological environment [30,31,32]. For example, in Inner Mongolia and Xinjiang [33], land use changes caused by resource over-exploitation have largely changed ecological processes [34]. In this context, investigating land use changes in open-pit coal mining areas can help optimize reclamation planning in such areas, adjust land use structure [35,36], and provide an important basis for the development of adequate management strategies and a sustainable coal mining policy.

As early as in the 1960s, studies on land use monitoring in mining areas have been carried out. In 1969, the land protection department in the United States monitored local mine environments and disasters, using remote sensing technology to monitor the land reclamation in coal mining areas, thereby providing a basis for the development of land reclamation strategies [37]. Brink et al. [38] took sub-Saharan Africa as the study area and, based on the high-scoring earth observation data, monitored and analyzed the changes in regional land use types during the period from 1975 to 2000. However, China’s remote sensing and geographic information system technology started late and is in a relatively undeveloped stage. The use of remote sensing satellites to monitor land use in mining areas was gradually developed after the 1980s. Since then, the remote sensing technology has gradually been developed, providing a certain amount of data for research and analysis.

Because of the late start of the monitoring technology, immature monitoring methods and low-accuracy monitoring results are frequent [39,40]. However, this phenomenon gradually decreases with the improvement of the research methods.

Globally, the application of remote sensing and GIS technology has gradually matured; various high-precision satellites, such as QuickBird, Landsat, Spot, and Sentinel, were born. The monitoring of land use in mining areas is performed with accurate data and technical support, which has resulted in a large number of studies. For instance, Raval et al. [41] used traditional remote sensing technology to monitor and quantitatively analyze land use change in kaolin mining areas in India from 2000 to 2009, providing technical support for the rapid mapping of land use changes in these areas. Sonter et al. [42] considered the mining area as a separate land use type for the classification of remote sensing images, described the land use change process in the Brazilian mining area over a period of time, and compared it with that of the surrounding non-mining areas, with the aim to analyze the differences and the underlying reasons. Using all archived Landsat imagery between 2000 and 2015, Wohlfart et al. [43] calculated the temporal and textual measures of spatially continuous spectra based on dense Landsat time series for each year to obtain values related to mining, agriculture, forestry and urbanization in the Yellow River Basin Zoning land cover change map. Using Landsat image data from 2013 to 2016, Padmanaban et al. [44] studied the land use change in a reclaimed mining area in Kirchheller Heide, Germany, using the vegetation cover index (NDVI) to analyze the changes in vegetation productivity and to determine the geological and surface environment changes that may occur in the mining area. Several methods of land use monitoring in mining areas have been developed, among which remote sensing and GIS technology are the most important ones and can be applied in high-precision and long-term land use monitoring in mining areas. However, the image processing flow of mining areas needs to be optimized, and the efficiency needs to be improved.

The application of remote sensing technology in the monitoring and management of land use change has gradually intensified. For most researchers interested in land-use change monitoring, the acquisition and processing of long-term remote sensing data are time-consuming and labor-intensive. When traditional image-processing software (ENVI, ERDAS) is used for land use change monitoring, original image data from specific channels need to be downloaded, and complex steps are required, such as image data correction, registration, splicing, and cropping. Processing power and storage space require researchers to have good theoretical knowledge and adequate image-processing skills. In this context, Google Earth Engine (GEE) [45] has become an important tool for geography and space-related research, providing powerful computing resources and massive online data. By invoking a large number of published geographic data products collected by the GEE platform and combining the algorithms provided by the researchers, online computing can be performed, which greatly reduces the workload of data acquisition and processing. More and more scholars use the GEE platform for land use monitoring research. For example, Hamud et al. [46] used the GEE platform to monitor land use cover changes in Somalia. Lin et al. [47] monitored land cover change on a rapidly urbanizing island using the GEE. This approach can greatly expand the time and space scale of their original research and provide national and even global research results [48,49,50]. The GEE platform makes up for the deficiency of traditional image-processing software and enriches the technical methods of land use monitoring research in mining areas.

Judging from the current global research progress, most of the current technologies are applied in small mining areas and are dominated by algorithm models. There are few studies on long-term, rapid, accurate and continuous land use classification in open-pit mining areas. In addition, most of the research is concentrated in a single mining area, and investigations on multiple mining areas of a specific mining area type are scarce, and the explanation of the driving factors behind land use evolution is insufficient. In this study, seven types of land use are investigated, namely cropland, forest, grassland, water body, mining land, residential/industrial land, and unused land, according to the present situation of land use in the open-pit mining areas in Inner Mongolia. Based on the emerging GEE platform, it solves the problems of difficult data collection, large data volumes, and low interpretation efficiency in long-term large-scale analyses. This study regards typical open-pit coal mines in Inner Mongolia as the research unit, and analyzes the dynamic evolution characteristics and driving factors of land use from 2001 to 2020. The main objectives are as follows: (i) to gain an in-depth understanding of the dynamic change in land use in open-pit coal mining areas in Inner Mongolia; (ii) to identify the causes of spatial changes in land use in typical open-pit coal mining areas in Inner Mongolia; (iii) to put forward policy suggestions on land exploitation and remediation in mining areas.

## 2. Study Area

**Figure 1:**
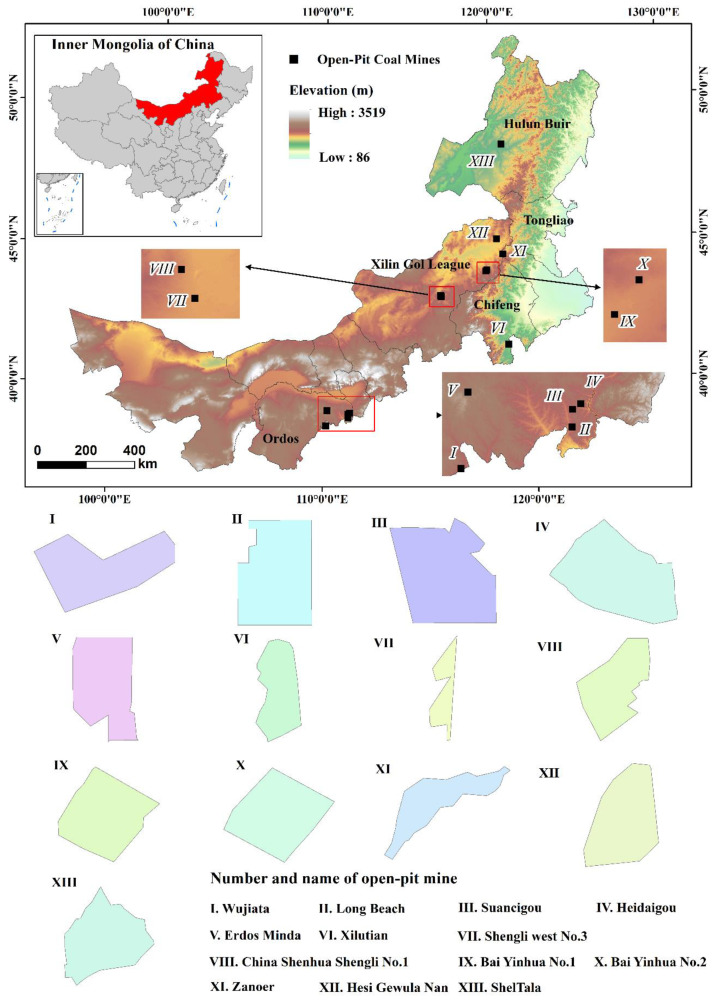
Locations and outlines of open-pit coal mines investigated in this study.

The Inner Mongolia Autonomous Region is located in northern China, at 37°24′–53°23′ north latitude and 97°12′–126°04′ east longitude (Figure 1), with a large plateau area, a large distance from the ocean, and mountains along the edge. The region has the richest mineral resources in China, with 17 kinds of mineral reserves in the forefront. However, high-intensity resource exploitation has a great impact on fragile ecosystems in the arid and semi-arid areas of the Inner Mongolia Autonomous Region. Open-pit mining has a more significant impact on the environment, such as ecosystem destruction and land resource degradation. Mining areas located in arid and semi-arid areas are particularly sensitive to this impact. Therefore, the land use change and ecological processes in the mining area are more complex and diverse, and its pattern change characteristics and laws are more representative.

The precise mining area is defined according to the mining rights, so we can only consider selecting open-pit coal mines according to the mining license information issued by the Ministry of Natural Resources. Moreover, to ensure a wider representation of mining areas, we need to ensure that open-pit coal mines that cover large, medium and small areas are covered. Therefore, we selected 13 open-pit coal mines as typical study areas in mining areas with relatively complete information (Figure 1). By connecting the original registered nodes of each mining area in sequence, the boundary mining area can be delineated. The open-pit coal mines are mainly distributed in Erdos, Xilin Gol League, Hulun beier and Chifeng, covering a total area of 391 km^2^ (the largest open-pit coal mine, Changtan, is 66.99 km^2^, whereas the smallest one, Shengli West No. 3, only covers 1.55 km^2^).

## 3. Data Sources and Methods

### 3.1. Data Sources

Generally speaking, 2001–2020 is an important period for the rapid development of China’s ecological protection and restoration and the innovation and transformation of its system and mechanism. This study used the 2001–2020 image data of Landsat on the GEE platform. Among them, mainly from April to September, GEE synthesized the image data and used them as a remote sensing data source combined with DEM data, the Chinese Academy of Sciences Resources and Environment Data Center [51] vegetation type data, vegetation zoning data, and meteorological data and the random forest model was used for land use classification.

According to the characteristics of the mining area, the land use types were divided into six categories, namely cropland, forest, grassland, water body, residential/industrial square land, mining land, unused land. Residential/industrial square land refers to residential and living ancillary facilities, industrial plants, and large industrial construction. Mining land refers to the mining, quarrying, sand mining (sand) fields, brick kilns and other ground production land and tailings dumps that are independent of residential areas. Unused land refers to tailing stacking land, bare land, bare rocks, and sand areas. The social and economic statistics of raw coal output used in this study were derived from the *Inner Mongolia Statistical Yearbook* [52], the *Regulations of Inner Mongolia Autonomous Region on Mineral Resources Management* [53], and the *Statistical Bulletin of National Economic and Social Development of Inner Mongolia Autonomous Region* [54].

### 3.2. Methods

#### 3.2.1. Dynamic Degree of Land Use

The dynamic degree of land use is based on the magnitude of land use change and represents the results of various types of area changes during the study period. It can directly reflect the change speed of different land use types and can be used to compare and analyze the change differences among various types [55]. In this paper, the dynamic degree model that reflects the absolute amount of land use change was used to monitor the speed change in each land use type in the study area, using the following equation:

(1) $$k_{j}=\frac{u_{b}-u_{a}}{u_{a}}\times \frac{1}{T}\times 100\%$$

where $k_{j}$ represents the dynamic degree of a certain land use type during the research period; $u_{a}$ represents the quantity of a certain land use type at the early stage of the research; $u_{b}$ represents the quantity of a certain land use type at the end of the research period; T represents the length of the research time.

The study of land use dynamic changes is an important approach to arrive at a deep understanding of the process of urban land use change, and is the main method to comprehend the evolution process and pattern of land use [56,57]. To deeply explore the land use change dynamic of typical open-pit mining areas in Inner Mongolia, the single land use dynamic degree method was used.

#### 3.2.2. Geographical Detector Model (GDM)

The GDM (geographical detector model) is an important method to detect the spatial pattern and genesis of geographic elements and is widely used in studies on land use driving mechanisms and climate change [58]. When importing the input data of GDM, discrete classification processing of driving factors is required in ArcGIS, and through sampling, discrete data of the dependent variables and result variables are obtained, and are finally imported into the GDM for factor analysis. The specific calculation method is as follows:

(2) $$q=1-\frac{1}{n\sigma^{2}}\sum_{i=1}^{m}n_{i}\times \sigma^{2}$$

where q is the explanatory power of the driving factor for the expansion of construction land; n is the total amount of driving factors, and $\sigma^{2}$ is the sample variance. The value range of q is (0, 1) and the larger the value, the stronger the explanatory power of the factor to land use change will be.

## 4. Results

### 4.1. Dynamic Evolution of Land Use in the Typical Open-Pit Coal Mine Area

#### 4.1.1. Land Use Pattern

By establishing polygon training samples of land use classification in GEE, a sample set of each corresponding land class was formed. Then, all the samples were fused into a sample set. Part of them were selected as training samples to participate in classification, and part of them were used as verification samples for precision verification. We used the random forest classifier in GEE, took training samples and images as input, and carried out supervised classification to obtain the raster data of land cover. After classification, the overall accuracy, kappa coefficient, and transfer matrix were calculated by using the verification data set and the classification results, and the accuracy was evaluated. When all the land use classification results meet the accuracy requirements (Table 1), the results were retained.

**Figure 2:**
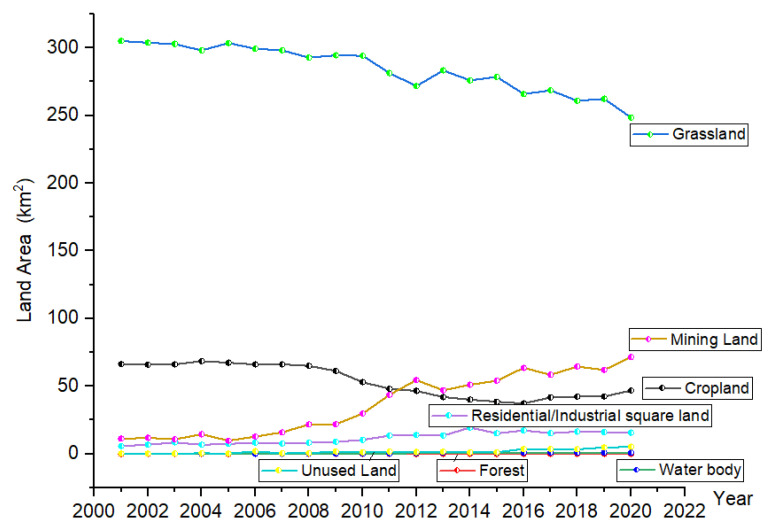
Land use types and areas in open-pit coal mines from 2001–2020.

From 2001 to 2020, the change trend of land use in open-pit coal mines was obvious (Figure 2), with large grassland and cropland areas and a considerable change range. The forest, water body, and unused land areas were small, and the fluctuation was relatively stable. The areas of mining land and residential/industrial square showed a fluctuating increase throughout the research period, whereas the grassland area showed a fluctuating decrease. The cropland area showed a downward trend from 2001 to 2015, followed by an increase after 2016. The mining land increased rapidly from 2006 to 2012, with a slower growth thereafter.

#### 4.1.2. Dynamic Degree of Land Use

According to the dynamic degree of land use in the study area (Table 2), from 2001 to 2020, the dynamic degree of unused land and mining land changed most significantly, accounting for 93.16% and 27.11%, respectively. Among them, from 2001 to 2005, the land with the largest change was residential/industrial square land, with a total transfer area of 1.68 km^2^ and a dynamic degree of 5.85%, followed by mining land with a transfer area of −1.42 km^2^ and a dynamic degree of −2.54%. From 2005 to 2010, the mining land showed the largest dynamic change, which was 16.97 km^2^, and the dynamic change degree was 26.53%, followed by cropland, whose change amount was −13.16 km^2^, with a dynamic degree of −3.98%. Compared with 2001–2005 and 2005–2010, in 2010–2015, the water body significantly changed the most, and the dynamic degree was 39.13%. From 2015 to 2020, the transfer of cropland kept increasing, with a change of 9.63 km^2^, and the dynamic degree increased to 5.17%; in contrast, the residential/industrial square land decreased by −1.72 km^2^, with a dynamic degree of −1.98%.

### 4.2. Spatial Driving Factor Analysis of Land Use Change in Typical Open-Pit Coal Mining Areas in Inner Mongolia

Identifying the causes of spatial changes in land use in typical open-pit coal mining areas in Inner Mongolia is of great significance for exploring the landscape ecological trends of land use changes, adjusting the industrial structure of mining areas, and arriving at sustainable land development [59,60]. From the perspective of spatial heterogeneity, this study uses the ArcGIS spatial analysis function to sample the spatial location and driving factors of land use change in the open-pit mining areas from 2001 to 2020 and used the GDM for q-value detection. From the perspective of time, this paper analyzes the impact of mining and land reclamation on land use change, as well as the impact of large-scale mining, reclamation and other activities on this change.

#### 4.2.1. Analysis of the Factors Influencing Mining and Reclamation in Mining Activities

**Figure 3:**
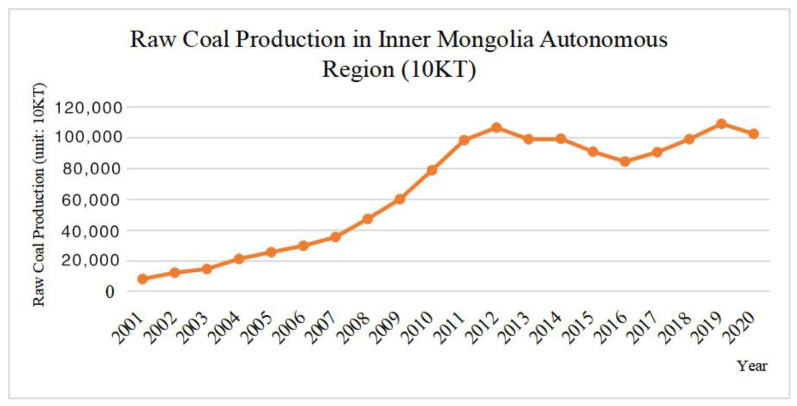
2001–2020 Raw coal production in Inner Mongolia Autonomous Region.

In the past 20 years, Inner Mongolia has witnessed large-scale mining activities. According to the Statistical Yearbook of Inner Mongolia and the Statistical Bulletin of National Economic and Social Development of Inner Mongolia Autonomous Region, from 2001 to 2020, the output of raw coal in the Inner Mongolia Autonomous Region showed a rapid growth, from 81.63 to 1025.51 million tons (Figure 3).

**Figure 4:**
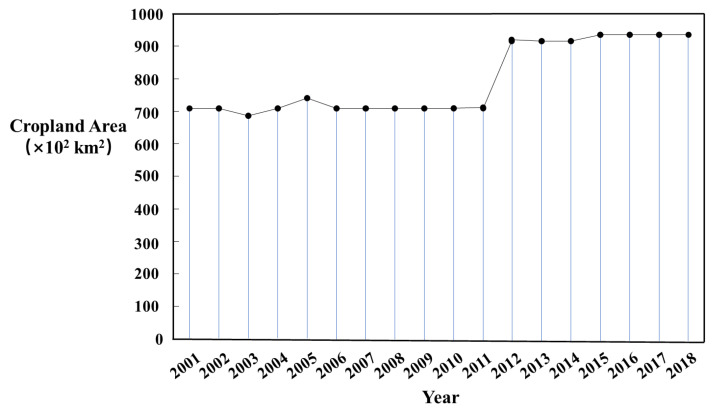
Changes in cropland area from 2001 to 2018 in the Inner Mongolia Autonomous Region.

Regarding the entire study period, the area of mining land showed a growth trend, which is closely related to the development of mining activities. From 2005 to 2012, the area of open field/unused land increased significantly, whereas that of cropland decreased greatly. After 2013, the area of mining land showed a fluctuating decrease, and some cropland recovered rapidly. This is due to the implementation of a number of land reclamation policies and measures in the study area from 2008 to 2020, which significantly improved the local land use structure. For example, in 2008 and 2013, the measures of the Inner Mongolia Autonomous Region for the management of mining and mineral deposits were issued and revised successively, and in 2009, the implementation plan for the management of mining the geological environment was issued. From 2009 to 2015, the Inner Mongolia Autonomous Region vigorously carried out the geological and ecological environment treatment in mining areas. In addition, according to the regulations on land reclamation issued by the State Council in 2011, the basic national policy of promoting mining enterprises to make rational use of land and implement cropland protection is one of the reasons for the overall increase in cropland after 2011. According to the Xinhua news agency, since 2007, the Inner Mongolia Autonomous Region has received a total of 53 national land consolidation projects, with a capital of CNY 350 million, for the transformation of wasteland, sandy land, and low-yield fields, resulting in 7713.3 hectares of new cropland. A total of 78 economic development zones were abolished in Inner Mongolia, and nearly 1333.3 hectares of land were restored. According to the Inner Mongolia Bureau of Statistics, from 2012 to 2018, the cultivated area increased by 163,000 hectares, with an average annual growth of about 0.3%. At the same time, in the grassland mining area, the land damage caused by mining has been effectively controlled (Figure 4). Therefore, mining activities and land reclamation can be regarded as the main reasons for land use changes in mining areas.

#### 4.2.2. Geographical Detector Model-Based Analysis of Natural and Geographic Drivers

To deeply explore the driving mechanisms of land use change in the open-pit coal mining area, the GDM was used to analyze the q-value and p-value of the open-pit coal mine, thereby determining the strength of the driving force. The results are shown in Table 3. The p-value of the eight spatial driving factors are all below 0.001, and the p-values of the distance from the urban road and the distance from the rural road are 0.06 and 0.81, respectively; the correlation between land use change in the open-pit coal mining area and the distance from urban roads and rural roads was weak. The distance from the national highway most significantly explained the land use change in the open-pit coal mining area, with a q-value of 0.19. The second most important factors were distance from the railway, the average annual temperature, average annual precipitation, distance from the county road and the elevation, and the q-values are 0.18, 0.16, 0.138, 0.12, and 0.12, respectively (Table 2).

By analyzing the driving factors of land use change in open-pit coal mines, we found that the most important factor for the spatial change in land use in open-pit coal mines is traffic location conditions, followed by climatic conditions and topographical conditions. Human activities have an important impact on land use changes and landscape patterns in mining areas. However, in recent years, the government has attached great importance to the monitoring and evaluation of the environmental impacts of mining, with the development of new technologies and optimized mining planning and design. Such efforts have resulted in the alleviation of the environmental destruction via open-pit coal mining, also showing that human factors can play a role in environmental protection through policy formulation and publicity [61]. In view of the land problem of open-pit coal mines, open-pit combined mining can be carried out in conditional mining areas, or the coordinated land-saving technology can be advocated, and the production management in the pre-mining planning stage, mining disturbance stage and layout recovery stage of the mining area should be strictly controlled to reduce the damage to the original landscape.

## 5. Discussion

### 5.1. Uncertainties

Land use classification based on remote sensing data is afflicted with certain errors due to various reasons, such as differences in evaluation accuracy. Therefore, in future research, the combination of remote sensing, RTK, UAV, and 3D laser scanning technology should be strengthened to improve the accuracy and quality of data extraction. This study conducted multi-party comparisons and on-the-spot investigations and tests to repeatedly demonstrate the compatibility of classification and calculation results with local conditions, with the aim to minimize the degree of error. This can ensure that the calculation results are credible and in line with natural, economic and social trends. At the same time, a variety of sampling methods (stratified sampling, probability statistics) can be explored to provide a test paradigm for the future research accuracy of remote sensing estimation of ecological assets in open-pit coal mining areas and to offer a technical basis for the formulation of ecological restoration goals in mining areas [62].

### 5.2. Comparison of the Mining Activities

Coal mining has led to major changes in land use in mining areas, and the ecological environment of mining areas has been affected and destroyed. Scholars from China and around the world have conducted numerous studies on land use identification, land space planning and reclamation, and ecosystem services in mining areas. For example, He et al. [16] proposed an improved model for identifying coal mine areas, which can monitor coal mining conditions in the mining area at any time. Gao et al. [15] studied the conflict of land use in the production-living ecological space of large-scale open-pit coal mines and proposed spatial planning optimization and land reclamation measures. Bian et al. [63] analyzed the change in ecosystem service value and characteristics on the basis of analyzing land use change in the mining area. With the rapid economic and social development, the irreplaceability of resource extraction will continue for a certain period [64]. These authors determined the impact of the mining and reclamation of metal mines, oil and gas fields, and coal bed methane and other mineral resources on land use and ecological effects and compared the evolution of different types of mineral mining on land use and landscape patterns. This paper also explores how the mining of different minerals affects the ecosystem, providing a scientific reference for the formulation of ecological restoration policies in mining areas. Future studies on metal mines (especially related to pollution), oil and gas fields, and coal bed methane (occupying large amounts of land) can provide a powerful reference for the coordinated development of the regional economy and the transformation of resource-exhausted cities.

### 5.3. Policy Significance

This paper studies the dynamic evolution of land use in open-pit coal mines from 2001 to 2020. The time span is large, reflecting the impact of open-pit coal mining on the structure and function of land use. However, some mining areas have not been fully exploited, especially some open-pit coal mines that were issued licenses and put into operation after 2017 and 2018. Due to the progress of the corresponding procedures in the mining areas, coal price adjustments in recent years, and even the impact of the epidemic, the mining progress has not been carried out as scheduled. Therefore, the life cycle scale research based on open-pit coal mining has certain limitations. Mineral resource development policies have a significant impact on land use changes in mining areas, and unified planning and management should be carried out in the following three stages: pre-mining planning, mining disturbance, and post-mining recovery. In future research, the entire life cycle of open-pit coal mines from the infrastructure construction period, mining period, reclamation period, stabilization (underground mining) period to the management and protection period can be explored. Carrying out comparative research at different scales, such as regional and mining site scales, can provide technical support for the development and use of mining areas and the formulation of ecological restoration policies in the later stage. Even after the cessation of mining activities, land resource degradation will still occur. In this sense, carrying out a comprehensive renovation of the whole life cycle of the mining area and improving the mechanism of mining environment compensation can be applied to achieve efficient and rational use of mining area land and to protect ecological integrity. We suggest that the coal mining subsidence areas and abandoned sand pits should be subjected to slope cutting and platform building, land leveling, and vegetation restoration, and measures such as the establishment of comprehensive enclosures and the sowing of grass seeds should be taken to restore the ecological environment of the mining area. At the same time, strengthening the management and protection work in the later stage and promoting the follow-up survival guarantee measures for shrubs, grass, shelter forests, and seedlings are important steps. In this context, it is crucial to investigate the environmental and geographical characteristics of the mining area and analyze the specific issues.

## 6. Conclusions

Based on remote sensing images, this study used the land use dynamics analysis and geographic detector model to explore the temporal evolution trend and driving factors of land use dynamics in typical open-pit coal mining areas in Inner Mongolia. In particular, we analyzed the dynamic change process of land use in typical open-pit coal mines from 2001 to 2020, identified the reasons for the spatial changes in land use, and put forward policy recommendations for the optimization of land mining and reclamation in mining areas. From 2001 to 2020, grassland, mining land, cropland, and residential/industrial square land dynamics significantly fluctuated the most, whereas the areas of forest, water body and unused land remained relatively stable. Mining activities and land reclamation were the main reasons for land use changes in the study area. The land use in the open-pit coal mining area is greatly affected by mining factors. From the perspective of spatial variation, the most important driving factor is the traffic location condition, followed by the climatic and topographical conditions. Land reclamation and ecological restoration in mining areas have a positive impact on land use change.

Multi-mineral, multi-scale, and long-term comprehensive studies on mining areas need to be performed in the future. Strengthening the comprehensive analysis of various methods, performing real dynamic simulation, and revealing the characteristics and internal mechanisms of the land use changes in the past can provide the theoretical foundation for future land use changes. At the same time, emphasis should be placed on strengthening the research on soil, vegetation restoration, and reconstruction methods in mining areas. Land reclamation and ecological reconstruction in mining areas should be guided by new technologies to promote the development of social, economic, and environmental benefits. By focusing on the improvement of ecological quality and ecological economic construction, measures such as land consolidation, forest restoration on abandoned mining land, and conservation forest planting in water resource areas can promote the comprehensive management of water and mining land ecosystems and improve regional ecosystem functions. Appropriate vegetation allocation modes should be selected for the configuration, planting, management, and protection of plants at different site types.

## Figures and Tables

**Table 1:** Overall accuracy and kappa coefficient values of land use classification.

Year	Kappa	Overall Accuracy	Year	Kappa	Overall Accuracy
2001	0.863	0.921	2011	0.861	0.921
2002	0.849	0.914	2012	0.852	0.915
2003	0.857	0.917	2013	0.845	0.912
2004	0.839	0.909	2014	0.868	0.924
2005	0.841	0.910	2015	0.866	0.923
2006	0.837	0.908	2016	0.903	0.944
2007	0.847	0.913	2017	0.884	0.933
2008	0.833	0.906	2018	0.849	0.914
2009	0.852	0.916	2019	0.859	0.919
2010	0.828	0.903	2020	0.854	0.916

**Table 2:** Dynamic degree of land use in open-pit coal mines (2001–2020).

Land Use Type		Cropland	Forest	Grassland	Water Body	Residential/Industrial Square Land	Mining Land	Unused Land
2001–2005	Variation (km^2^)	1.12	0.00	−1.38	0.00	1.68	−1.42	0.00
	Dynamic Degree k(%)	0.34	0.00	−0.09	−0.34	5.85	−2.54	0.07
2005–2010	Variation (km^2^)	−13.16	0.00	−5.20	0.11	2.09	16.97	−0.82
	Dynamic Degree k(%)	−3.98	−2.50	−0.35	14.73	5.04	26.53	−8.33
2010–2015	Variation (km^2^)	−9.39	0.02	−2.90	0.65	1.64	10.45	−0.47
	Dynamic Degree k(%)	−3.91	30.77	−0.21	39.13	2.42	4.79	−5.56
2015–2020	Variation (km^2^)	9.63	0.02	−17.44	−0.11	−1.72	8.03	1.59
	Dynamic Degree k(%)	5.17	13.79	−1.31	−2.28	−1.98	2.53	8.70
2001–2020	Variation (km^2^)	−19.48	0.02	−56.51	0.72	9.88	60.42	4.98
	Dynamic Degree k(%)	−1.47	5.21	−0.93	22.60	8.58	27.11	93.16
	Annual Change (km^2^)	−0.97	0.00	−2.83	0.04	0.49	3.02	0.25

Note: The data in the table were calculated according to the interpreted land use data and Formula (1).

**Table 3:** q-values of land use changes in open-pit coal mines.

Detection Type	x_1_	x_2_	x_3_	x_4_	x_5_	x_6_	x_7_	x_8_	x_9_	x_10_
q value	0.02	0.12	0.19	0.12	0.14	0.08	0.18	0.16	0.138	0.01
p value	0.06	≤0.001	≤0.001	≤0.001	≤0.001	≤0.001	≤0.001	≤0.001	≤0.001	0.81

Note: x_1_ represents the distance from the city road; x_2_ represents the elevation; x_3_ represents the distance from the national highway; x_4_ represents the average annual precipitation; x_5_ represents the distance from the provincial road; x_6_ represents the slope; x_7_ represents the distance from the railway; x_8_ represents the annual average temperature; x_9_ represents the distance from the county road, and x_10_ represents the distance from the township road.

## Data Availability

All relevant data sets in this study are described in the manuscript.
